# The Brain Metabolic Correlates of the Main Indices of Neuropsychological Assessment in Alzheimer’s Disease †

**DOI:** 10.3390/jpm10020025

**Published:** 2020-04-18

**Authors:** Agostino Chiaravalloti, Maria Ricci, Daniele Di Biagio, Luca Filippi, Alessandro Martorana, Orazio Schillaci

**Affiliations:** 1 Department of Biomedicine and Prevention, University Tor Vergata, 00133 Rome, Italy; agostino.chiaravalloti@uniroma2.it (A.C.); orazio.schillaci@uniroma2.it (O.S.); 2 IRCCS Neuromed, 86077 Pozzilli, Italy; 3 UOC Nuclear Medicine, Policlinico Tor Vergata, 00133 Rome, Italy; danieledibiagio@gmail.com; 4 UOC Nuclear Medicine, Santa Maria Goretti Hospital, 04100 Latina, Italy; lucfil@hotmail.com; 5 UOSD Centro Demenze Policlinico Tor Vergata, 00133 Rome, Italy; martorana@med.uniroma2.it; 6 Department of System Medicine, University Tor Vergata, 00133 Rome, Italy

**Keywords:** Alzheimer’s disease, PET/CT, (18F)FDG, neuropsychological assessment

## Abstract

Background: The study aimed to investigate the relationships between F-18 fluorodeoxyglucose (18F)FDG uptake and neuropsychological assessment in Alzheimer’s disease (AD). Methods: We evaluated 116 subjects with AD according to the NINCDS-ADRDA criteria. All the subjects underwent a brain PET/CT with (18F)FDG, cerebrospinal fluid (CSF) assay, mini-mental state examination (MMSE) and further neuropsychological tests: Rey auditory verbal learning test, immediate recall (RAVLT immediate); Rey auditory verbal learning test, delayed recall (RAVLT, delayed); Rey complex figure test, copy (RCFT, copy); Rey complex figure test, delayed recall (RCFT, delayed); Raven’s colored progressive matrices (RCPM); phonological word fluency test (PWF) and Stroop test. We performed the statistical analysis by using statistical parametric mapping (SPM12; Wellcome Department of Cognitive Neurology, London, UK). Results: A significant relationship has been reported between (18F)FDG uptake and RAVLT immediate test in Brodmann area (BA)37 and BA22 and with RCFT, copy in BA40, and BA7. We did not find any significant relationships with other tests. Conclusion: In the AD population, brain (18F)FDG uptake is moderately related to the neuropsychological assessment, suggesting a limited impact on statistical data analysis of glucose brain metabolism.

## 1. Introduction

Subjective memory deficit, together with evidence of memory decline and cognitive impairment during the past months or few years, are key features for Alzheimer’s disease (AD) already at the prodromal (or mild cognitive impairment; MCI) stage [1]. Although cognitive tests are frequently used as outcome measures in clinical trials, there are a number of limitations associated with their use [2].

Neurodegenerative AD-related changes are known to accumulate progressively 15 to 20 years before dementia, and even years before the detection of clinical deficits [3]. Cognitive decline in AD, generally assessed by neuropsychological battery [4], is postulated as consequential from neurological pathology as portrayed by biomarkers [5], but the specificity of these associations is still a matter of debate [6]. Particularly, the correspondence between the neuropsychological assessment and standard neuroimaging biomarkers remains incomplete, and the relationship between neuropsychological performance and individual variation in clinical AD neuroimaging markers is likely to be both convergent and unique [5,7]. Although it is tempting to presume neurological substrates causing poor performance at a common neuropsychological assessment, the substrates underlying the cognitive process may be reorganized in the diseased brain of patients with MCI, possibly due to neurodegenerative changes or functional compensation. It is important to understand how these biomarkers, interact to influence cognitive change to isolate the combination of pathologies that contribute most to decline, in order to inform the clinical utility and validation of cognitive tests [5] and the neuroimaging correlates to test performance. 

In particular, data concerning the relationship between glucose metabolism and common neuropsychological assessment are poor, even if the results of the neuropsychological tests may be considered as statistical parameters in research papers that include brain data analysis, by using computer-aided metrics such as statistical parametric mapping (SPM) [8,9].

The present study aimed to evaluate the brain metabolic correlates of the main indices of neuropsychological assessment by studying their relationships to cortical and subcortical F-18 fluorodeoxyglucose (18F)FDG uptake in a cohort of subjects with AD. Moreover, this paper aimed to evaluate the impact of neuropsychological assessment as parameters in SPM brain data analysis. For this purpose, all the subjects underwent a brain PET/CT scan using (18F)FDG, a complete neuropsychological assessment that included mini-mental state examination (MMSE). Moreover, the following indices of neuropsychological assessment were determined in the AD population: Rey auditory verbal learning test, immediate recall (RAVLT immediate); Rey auditory verbal learning test, delayed recall (RAVLT, delayed); Rey complex figure test, copy (RCFT, copy); Rey complex figure test, delayed recall (RCFT, delayed); Raven colored progressive matrices (RCPM); phonological word fluency test (PWF) and Stroop test. A cerebrospinal fluid (CSF) assay for amyloid, total tau, and phosphorylated tau was performed in all patients.

An initial version of this paper was presented as a conference paper at the 29th European Association of Nuclear Medicine Congress.

## 2. Materials and Methods

We evaluated 116 subjects with a new clinical diagnosis of AD (males = 66; females = 50) according to the NINCDS-ADRDA criteria [10]. The mean age was 71.4 ± 6 years old. A complete clinical investigation was performed in all patients, including medical history, mini-mental state examination (MMSE), a complete blood screening (including routine exams, thyroid hormones, and level of B12). Moreover, in all patients, a neurologist examination, neuropsychological examination, a complete neuropsychiatric evaluation, and neuroimaging consisting of magnetic resonance imaging (1.5 T MRI) was performed. Exclusion criteria were the following: isolated deficits and/or unmodified MMSE (<25/30) on revisit (6, 12, and 18 months follow-up), clinically manifest acute stroke in the last 6 months (Hachinsky scale >4, and radiological evidence of subcortical lesions), as described in previous papers [11,12,13]. None of the patients enrolled had pyramidal and/or extrapyramidal signs reported at the neurological examination. At the time of enrollment, in the 30 days before participating in this study, none of the patients had been treated with drugs that might have modulated cerebral cortex excitability, including antidepressants, neuroactive drugs (i.e., benzodiazepines, antiepileptic drugs or neuroleptics), or cholinesterase inhibitors. The study was performed according to the Declaration of Helsinki and approved by the local ethics committee of the Tor Vergata University in Rome. All participants or their legal guardians gave the written informed consent after receiving an extensive disclosure of the study. A cognitive profile consistent with mild dementia (according to neuropsychological evaluation, including the MMSE) has been described in all AD patients.

In order to improve the diagnostic accuracy of the AD patients, a lumbar puncture and CSF sampling was performed in all patients. The first 12 mL of CSF was collected in a polypropylene tube, then directly transported to the local laboratory for centrifugation at 2000× *g* at +4 °C for 10 min. The supernatant was pipetted off, gently stirred and mixed to avoid potential gradient effects, and aliquoted in 1 mL portions in polypropylene tubes that were stored at −80 °C pending biochemical analyses, without being thawed and re-frozen. Then, CSF total Tau (T-Tau) and phosphorylated Tau (Thr181, p-Tau) concentration was evaluated using a sandwich ELISA (Innotest hTAU-Ag, Innogenetics, Gent, Belgium). CSF Aβ1–42 levels were determined using a sandwich ELISA (Innotest^®^ ß- amyloid (1–42), Innogenetics, Gent, Belgium), specifically elaborated to measure Aβ containing both the first and 42nd amino acid, as described in previous papers [11,12,13,14,15]. 

All the subjects underwent a brain PET/CT scan using (18F)FDG, mini-mental state examination (MMSE). Several indices of neuropsychological assessment were explored: Rey auditory verbal learning test, immediate recall (RAVLT immediate); Rey auditory verbal learning test, delayed recall (RAVLT, delayed); Rey complex figure test, copy (RCFT, copy); Rey complex figure test, delayed recall (RCFT, delayed); Raven’s colored progressive matrices (RCPM); phonological word fluency Test (PWF) and Stroop test. The relationship between brain uptake of (18F)FDG and CSF biomarkers were analyzed using statistical parametric mapping (SPM12; Wellcome Department of Cognitive Neurology, London, UK) implemented in Matlab R2018a using sex, age, and CSF biomarkers as covariates.

### 2.1. PET/CT Scanning

The PET/CT system Discovery VCT (GE Medical Systems, Tennessee, TN, USA) has been used to assess (18F)FDG brain distribution in all patients using a 3D-mode standard technique in a 256 *×* 256 matrix. Reconstruction was performed using the 3-dimensional reconstruction method of ordered-subsets expectation maximization (OSEM) with 20 subsets and with 4 iterations. The system combines a high-speed ultra 16-detector-row (912 detectors per row) CT unit and a PET scanner with 10,080 bismuth germanate crystals in 24 rings (axial full width at half-maximum 1 cm radius, 5.2 mm in 3D mode, an axial field of view 157 mm). A low-amperage CT scan of the head for attenuation correction (40 mA; 120 Kv) was performed before PET image acquisition. All subjects fasted for at least 5 h before intravenous injection of (18F)FDG ; the serum glucose level was up to 95 mg/mL in all of them. All the subjects were injected intravenously with 185–210 MBq of (18F)FDG and hydrated with 500 mL of saline (0.9% sodium chloride). The scan started 30 min after the injection in all the subjects according to standard guidelines, as described in previous papers [13,16,17].

### 2.2. Statistical Analysis

Differences in brain (18F)FDG uptake were analyzed using statistical parametric mapping (SPM12, Wellcome Department of Cognitive Neurology, London, UK) implemented in Matlab R2018a (Mathworks, Natick, Massachusetts, USA). PET data were subjected to affine and nonlinear spatial normalization into the MNI space. Then, the spatially set of images was smoothed with an 8 mm isotropic Gaussian filter to blur individual variations in gyral anatomy and to increase the signal-to-noise ratio. Images were globally normalized using proportional scaling to remove confounding effects to global CBF changes, with a threshold masking of 0.8. The statistical parametric maps were transformed into a normal distribution unit. Correction of SPM coordinates to match the Talairach coordinates was achieved by the subroutine implemented by Matthew Brett (http://www.mrc-cbu.cam.ac.uk/Imaging). Brodmann areas (BA) were then identified at a range of 0 to 3 mm from the corrected Talairach coordinates of the SPM output isocentres, after importing them by Talairach client (http://www.talairach.org/index.html). A statistical height threshold was equal to or lower than *p* < 0.001 at both clusters, and voxel-level was accepted as significant. We considered significant a cluster extension of more than 125 (5 × 5 × 5 voxels, i.e., 11 × 11 × 11 mm) contiguous voxels, based on the calculation of the partial volume effect resulting from the spatial resolution of the PET camera (about the double of full width at half maximum), as described in previous papers of the same research group [13].The resulting SPM data was correlated to each index of neuropsychological assessment, in order to study their relationships to cortical and subcortical (18F)FDG uptake.

## 3. Results

The values of CSF amyloid, total tau, and phosphorylated tau were respectively 363.6 ± 162, 689 ± 338.1, and 92.4 ± 70.7 pg/mL. A general overview of the patient population is reported in Table 1. Most of the cases were sporadic, but in about 15% of enrolled patients, there was a family link.

Neuropsychological assessment resulted in 22.6 ± 8.6 for RAVLT, immediate; 71.4 ± 5,9 for RAVLT, delayed; 18.2 ± 10.4 for RCFT, copy; 7.6 ± 6 for RCFT, delayed; 18.8 ± 9 (RCPM); 22.2 ± 10.1 for PWF and 44.6 ± 36.2 for Stroop test (Table 2).

**Figure 1:**
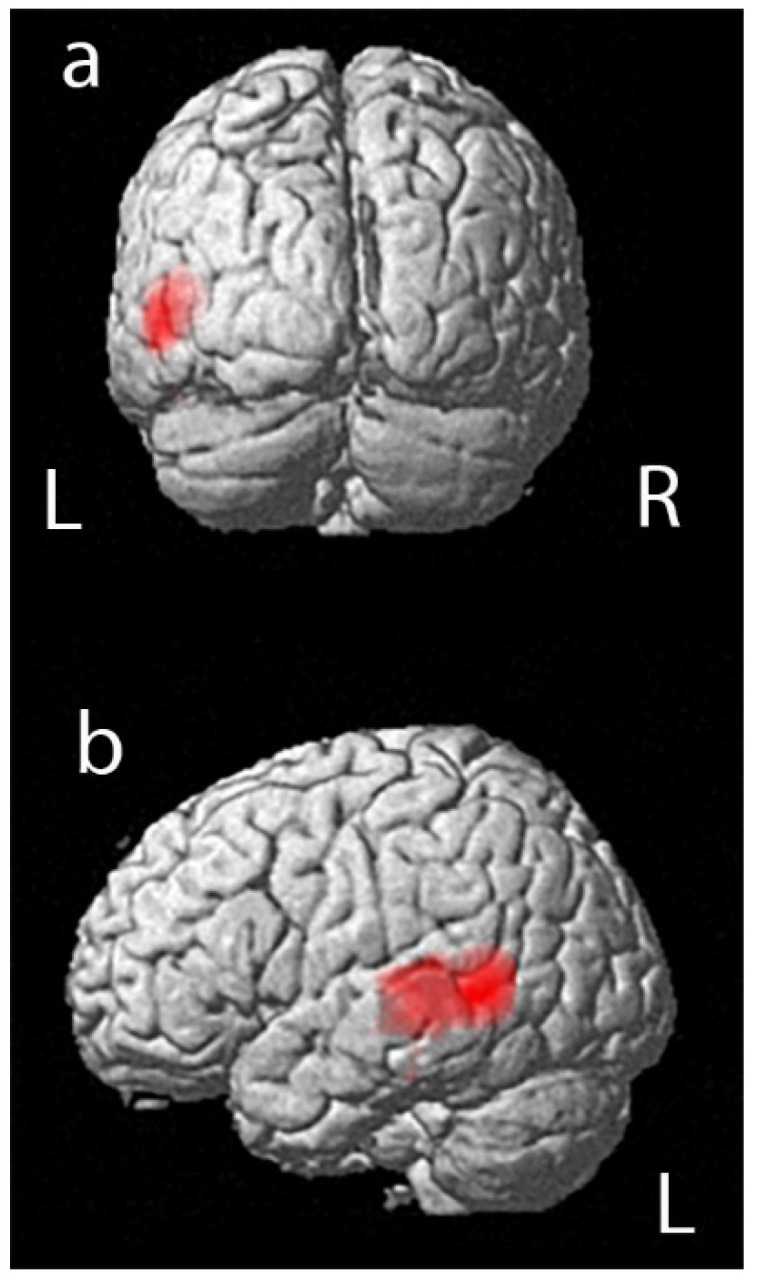
3D rendering of the results of statistical parametric mapping analyses between (18F)FDG uptake and performance in RAVLT immediate that shows a positive correlation in a large portion of the left temporal lobe (Brodmann area 37 and Brodmann area BA22): (**a**) posterior view; (**b**) left lateral view.

A positive correlation was reported in the statistical analysis between the PET data and performance in RAVLT immediate. We found a significant relationship between (18F)FDG uptake and performance in RAVLT immediate in a large portion of the left temporal lobe, in a cluster of 1141 voxels that included left temporal middle and superior gyrus, as shown in Figure 1 and Table 3 (positive correlation in Brodmann area 37 and Brodmann area 22).

**Figure 2:**
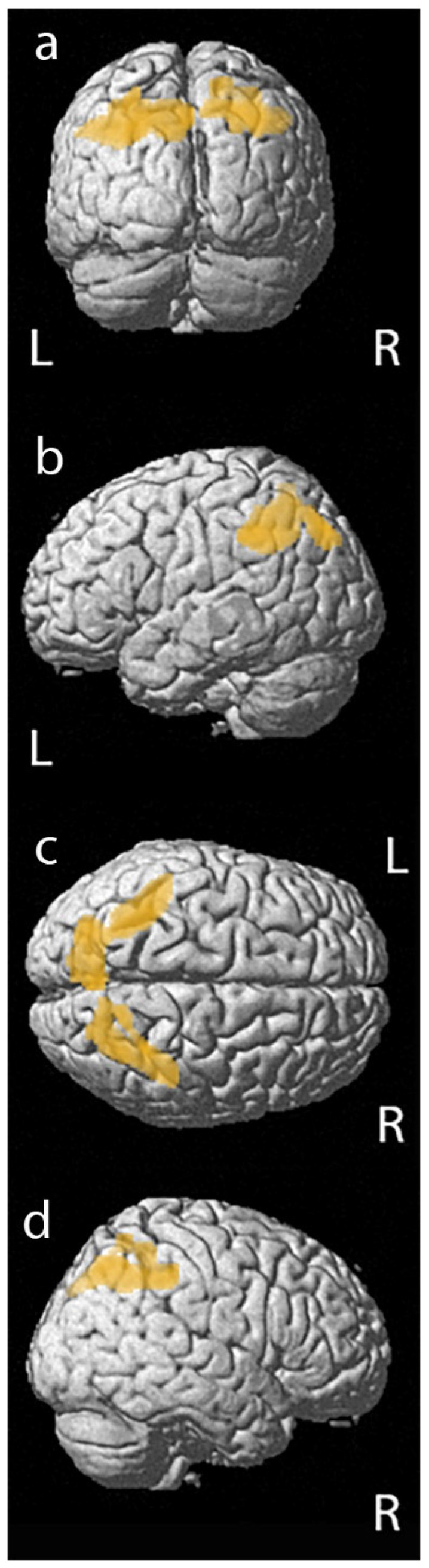
3D rendering of the results of statistical parametric mapping analyses between (18F)FDG uptake and performance in RCFT copy that shows a positive correlation in left and right Brodmann area 40 and left and right Brodmann area 7: (**a**) posterior view; (**b**) left lateral view; (**c**) superior view; (**d**) right lateral view.

Furthermore, a significant correlation was described between the PET data and performance in RCFT, copy. We found a significant positive relationship between (18F)FDG uptake and performance in RCFT, copy in a cluster of 1177 voxels that included left parietal inferior lobule and precuneus, and in a cluster of 804 voxels that include right parietal superior and inferior lobules (left and right BA40 and left and right BA7), as reported in Figure 2 and Table 4. We did not find any significant relationships between (18F)FDG uptake and other tests performed.

## 4. Discussion

Cognitive performance is an important outcome measure in AD diagnosis and progression. Nevertheless, the symptomatic significance of improvement or decline in clinical tests has not been well established, making it difficult to set a standard for what is meant by meaningful improvement [6]. Cognitive decline in AD is generally assessed by a neuropsychological battery [4]. An optimal outcome measure would reflect clinically-significant patient function, provide reliable measurements with minimal variability, and track a physiologically relevant disease process. However, the cognitive assessment also has several limitations, such as a large inter and intraindividual variability and floor and ceiling effects [2].

The neuropsychological test results may be considered as statistical parameters in research papers that include brain data analysis by using computer-aided metrics, such as statistical parametric mapping (SPM) [8]. For each cognitive measure, difference scores were entered into a regression analysis in SPM as the independent variable [9] or as a covariate.

The goal of the present study was to examine the brain metabolic correlates of the main indices of neuropsychological assessment tests in order to evaluate their impact on SPM brain data analysis.

We found a significant relationship between (18F)FDG uptake and performance in RAVLT immediate in a large portion of the left temporal lobe (positive correlation in BA37 and BA22) and with RCFT, copy (positive correlation in left and right BA40 and left and right BA7). We did not find any significant relationships with other tests.

A previous paper evaluated the brain metabolic correlates of the main indices of RAVLT, showing a significant correlation between the delayed recall score and metabolism in the posterior cingulate gyrus of both hemispheres and left precuneus, as well as between a score of long-term percent retention and metabolism in the left posterior cingulate gyrus, precuneus, and orbitofrontal areas. No correlation was found between immediate or total recall scores and glucose metabolism [1]. Nevertheless, the sample analyzed was a group of 54 elderly subjects with memory complaints. Therefore these differences, with our results, may be both due to the different sized sample and to the worse clinical conditions of our patients (elderly subjects with memory complaints vs. clinically diagnosed AD) with subsequent cortical hypometabolism according to the AD pattern. Neurological substrates underlying the tests widely used, such as delayed recall and executive function, were explored in MCI, whereas it was only partially explored in diagnosed AD. 

A paper concerning MCI performances on delayed recall and executive function suggested that hypometabolism in the right medial temporal cortex, right prefrontal cortex, left superior parietal cortex, and bilateral posterior cingulate reflect impairments in delayed recall, while hypometabolism in the right prefrontal cortex mirrors deficits in executive function in MCI [18]. The differences with our results may be due to differences in both the methods and in the stage of disease of the examined population. A further paper evaluated the relationships of FDG-PET metabolism with cognition in MCI: the composite score predicted variation in cortical metabolism, and TMT B was significantly correlated with PET metabolism. The results indicated that RAVLT and TMT B are sensitive to variation in AD neuroimaging markers in MCI but, contrary to our paper focused on SPM brain analysis; the Statistical Package for the Social Sciences (SPSS version 22.0) was used [5]. Even if both statistical analysis and enrolled populations are different from our methods, these findings partially support our results concerning the correlation of RAVLT test performance with FDG uptake in AD.

Furthermore, a previous study compared glucose metabolism and clinical measurements, reporting that baseline and longitudinal FDG-ROI measures are sensitive to poor performance at neuropsychological assessment and validate the cognitive and functional relevance of longitudinal changes in (18F)FDG measurement. Nevertheless, the indices used in this paper were The Functional Activities Questionnaire (FAQ) and Alzheimer’s Disease Assessment Scale—cognitive subscale (ADAS-cog) [6].

The patients and methods applied in the literature are as heterogeneous as the results obtained. According to our results concerning diagnosed AD, glucose metabolism of the left temporal lobe correlated to RAVLT, whereas glucose metabolism of the bilateral parietal lobe correlated to RCFT, copy. We did not find any significant relationships with other tests. Therefore, cortical and subcortical glucose consumption appear moderately related to the neuropsychological assessment. Even if the clinical usefulness of neuropsychological assessment is well established, our results suggest a limited impact on the data analysis of brain metabolism in patients with AD. Therefore, we suggest using these parameters in the statistical analysis of neuroimaging biomarkers by dedicated software, especially FDG uptake, just in the particular analysis concerning specific clinical aspects. Generally, considering the heterogeneous data about the influence of neuropsychological assessment on PET data, the use of neuropsychological indices as a covariate or independent variables in SPM analysis can be avoided in these patients.

## Figures and Tables

**Table 1:** A general overview of age, mini-mental state examination (MMSE), and Cerebral spinal fluid (CSF) parameters in the whole population.

Whole Population (*n* = 116)	Mean ± SD (Range)
Age (years)	71.4 ± 6 (48–82)
MMSE	20.3 ± 4.9 (9–30)
Amyloid (pg/mL)	363.6 ± 162 (70–913)
T-Tau (pg/mL)	689 ± 338.1 (173–1749)
P-Tau (pg/mL)	92.4 ± 70.7 (34–661)

**Table 2:** A general overview of Neuropsychological tests results in the whole population: Rey auditory verbal learning test, immediate recall (RAVLT immediate); Rey auditory verbal learning test, delayed recall (RAVLT, delayed); Rey complex figure test, copy (RCFT, copy); Rey complex figure test, delayed recall (RCFT, delayed); Raven’s colored progressive matrices (RCPM); phonological word fluency test (PWF) and Stroop test.

Neuropsychological Test	Mean ± SD (Range)
RAVLT immediate	22.6 ± 8.6 (0–43)
RAVLT delayed	71.4 ± 5.9 (48–82)
RCFT copy	18.2 ± 10.4 (0–36)
RCFT delayed	7.6 ± 6 (0–26)
RCPM	18.8 ± 9 (0–36)
PWF	22.2 ± 10.1 (0–55)
Stroop Test	44.6 ± 36.2 (0–162)

**Table 3:** A general overview of statistical parametric mapping analyses that detected a positive correlation in Brodmann area (BA) 37 and Brodmann area 22 of FDG uptake and performance in RAVL immediate test. The Brodmann areas were then identified at a range of 0 to 3 mm from the corrected Talairach coordinates of the SPM output isocentres. A statistical height threshold was equal to or lower than *p* < 0.001 at both clusters, and voxel-level was accepted as significant. We considered as significant a cluster extension of more than 125 contiguous voxels.

Analysis	Cluster Level					Voxel Level	
Positive Correlation	p(FWE-corr)	q(FDRcorr)	Extent	Cortical Region	Z Score of Maximum	Talairach Coordinates	Cortical Region
	0.003	0.007	1141	Left temporal, middle temporal gyrus	4.55	−48, −52, 4	BA 37
				Left temporal, middle temporal gyrus	3.68	−54, −36, 8	BA 22
				Left temporal, superior temporal gyrus	3.60	−56, −28, 6	BA 22

**Table 4:** A general overview of statistical parametric mapping analyses that detected a positive correlation in left and right Brodmann area (BA) 7 and left and right Brodmann area 40 of FDG uptake and performance in RCFT, copy test. The Brodmann areas were then identified at a range of 0 to 3 mm from the corrected Talairach coordinates of the SPM output isocentres. A statistical height threshold was equal to or lower than *p* < 0.001 at both clusters, and voxel-level was accepted as significant. We considered as significant a cluster extension of more than 125 contiguous voxels.

Analysis	Cluster Level					Voxel Level	
Positive Correlation	p(FWE-corr)	q(FDRcorr)	Extent	Cortical Region	Z Score of Maximum	Talairach Coordinates	Cortical Region
	0.002	0.005	1177	Left parietal, inferior parietal lobule	4.84	−34, −50, 36	BA 40
				left parietal, precuneus	4.25	−24, −76, 42	BA 7
				left parietal, precuneus	4.08	−16, −74, 48	BA 7
	0.011	0.013	804	Right parietal, superior parietal lobule	4.52	34, −56, 48	BA 7
				right parietal, superior parietal lobule	3.90	28, −66, 48	BA 7
				right parietal, inferior parietal lobule	3.70	42, −40, 42	BA 40
